# Associations between the neighbourhood food environment, neighbourhood socioeconomic status, and diet quality: An observational study

**DOI:** 10.1186/s12889-016-3631-7

**Published:** 2016-09-15

**Authors:** Maria McInerney, Ilona Csizmadi, Christine M. Friedenreich, Francisco Alaniz Uribe, Alberto Nettel-Aguirre, Lindsay McLaren, Melissa Potestio, Beverly Sandalack, Gavin R. McCormack

**Affiliations:** 1 Department of Community Health Sciences, Cumming School of Medicine, University of Calgary, 3280 Hospital Drive, North West Calgary, Alberta T2N 4Z6 Canada; 2 Department of Cancer Epidemiology and Prevention Research, CancerControl Alberta, Alberta Health Services, Calgary, Canada; 3 Department of Oncology, Cumming School of Medicine, University of Calgary, Calgary, Canada; 4 The Urban Lab, Faculty of Environmental Design, University of Calgary, PF 3201-2500 University Drive NW, Calgary, Alberta T2N 1 N4 Canada; 5 Department of Pediatrics, University of Calgary, Calgary, Canada; 6 Research Institute for Child and Maternal Health, Alberta Children’s Hospital, Calgary, Canada; 7 Alberta Cancer Prevention Legacy Fund, Alberta Health Services, Calgary, Canada

**Keywords:** Diet quality, Neighbourhood, Built environment, Food environment, Socioeconomic status

## Abstract

**Background:**

The neighbourhood environment may play an important role in diet quality. Most previous research has examined the associations between neighbourhood food environment and diet quality, and neighbourhood socioeconomic status and diet quality separately. This study investigated the independent and joint effects of neighbourhood food environment and neighbourhood socioeconomic status in relation to diet quality in Canadian adults.

**Methods:**

We undertook a cross-sectional study with *n* = 446 adults in Calgary, Alberta (Canada). Individual-level data on diet and socio-demographic and health-related characteristics were captured from two self-report internet-based questionnaires, the Canadian Diet History Questionnaire II (C-DHQ II) and the Past Year Physical Activity Questionnaire (PAQ). Neighbourhood environment data were derived from dissemination area level Canadian Census data, and Geographical Information Systems (GIS) databases. Neighbourhood was defined as a 400 m network-based ‘walkshed’ around each participant’s household. Using GIS we objectively-assessed the density, diversity, and presence of specific food destination types within the participant’s walkshed. A seven variable socioeconomic deprivation index was derived from Canadian Census variables and estimated for each walkshed. The Canadian adapted Healthy Eating Index (C-HEI), used to assess diet quality was estimated from food intakes reported on C-DHQ II. Multivariable linear regression was used to test for associations between walkshed food environment variables, walkshed socioeconomic status, and diet quality (C-HEI), adjusting for individual level socio-demographic and health-related covariates. Interaction effects between walkshed socioeconomic status and walkshed food environment variables on diet quality (C-HEI) were also tested.

**Results:**

After adjustment for covariates, food destination density was positively associated with the C-HEI (β 0.06, 95 % CI 0.01-0.12, *p* = 0.04) though the magnitude of the association was small. Walkshed socioeconomic status was not significantly associated with the C-HEI. We found no statistically significant interactions between walkshed food environment variables and socioeconomic status in relation to the C-HEI. Self-reported physical and mental health, time spent in neighbourhood, and dog ownership were also significantly (*p* < .05) associated with diet quality.

**Conclusions:**

Our findings suggest that larger density of local food destinations may is associated with better diet quality in adults.

**Electronic supplementary material:**

The online version of this article (doi:10.1186/s12889-016-3631-7) contains supplementary material, which is available to authorized users.

## Background

Poor diet quality is associated with malnutrition and chronic conditions including obesity and overweight, cardiovascular diseases, hypertension, diabetes mellitus, some cancers, and adverse mental health [1, 2]. Diet quality reflects multiple dimensions of the nutritional composition of a diet including: the diversity of foods (variety); the sufficiency of nutrient intake and adherence to national dietary recommendations (nutrient adequacy); whether certain nutrients are consumed in excess or in moderation, and; the overall balance of foods and nutrients [3]. Despite improvements in nutrient adequacy, excess consumption of nutrient-poor and energy-dense foods has resulted in an overall decrease in diet quality globally over the past 20 years [4].

Determinants of diet quality are multi-level and include intra-individual, inter-individual, physical environmental, policy-related, and cultural factors [5, 6]. Globally, many studies have examined the associations between diet (namely fruit and vegetable and fast-food consumption), the neighbourhood food environment [7–9], and neighbourhood socioeconomic environment [10–12]. Studies investigating relations between the objectively-assessed neighbourhood food environment and diet have typically relied on Geographical Information Systems (GIS)-derived measures proximity and availability of ‘healthy food destinations’ (e.g. supermarkets) and ‘unhealthy food destinations’ (e.g., fast-food restaurants, convenience stores) [8, 13]. In particular, studies often focus on the density of, and proximity to, supermarkets, convenience stores or fast-food restaurants from home address in relation to fruit and vegetable and fast-food consumption [7, 8]. However, it is challenge to designate food destinations as ‘healthy’ versus ‘unhealthy’ using spatial data given that most food destinations provide opportunity to purchase a variety of food types [14]. Furthermore, while fruits, vegetables and fast-food are important components of a diet, these indicators alone do not provide a comprehensive measure of diet quality (i.e., variety, adequacy, moderation, and balance).

Few studies to date have examined the objectively-assessed neighbourhood food environment in relation to multidimensional measures of diet quality [7, 8]. Notably only one Canadian study [15] has previously examined the neighbourhood food environment in relation to diet quality in adults. This study found no associations between GIS-assessed density, diversity, proximity of food destinations, or ratio of supermarkets to convenience and fast-food restaurants within the neighbourhood, and diet quality, measured using the Canadian Health Eating Index (C-HEI) [15]. Further, there is substantial variation in the magnitude and direction of associations found between the neighbourhood food environment and diet in studies undertaken internationally [9, 16–19]. The mixed findings suggest that there may be alternative explanations for neighbourhood differences in diet aside from density of, and, proximity to ‘healthy’ and ‘unhealthy’ food destinations [7]. For example, the number of all food destinations (regardless of ‘healthy’ versus ‘unhealthy’) in a neighbourhood may be important for diet quality as a result of a concept referred to as “gains from variety” [20, 21] which stipulates that a greater number of destinations often lends increased variety, competitive price, and improved access of products available. Thus, the number of food destinations in a neighbourhood may impact food variety, price, and accessibility which could contribute to differences in diet quality. The mixed findings, as well as the lack of Canadian-based evidence, suggest that more research on the associations between the neighbourhood food environment and diet quality is needed.

At the individual [22, 23] and neighbourhood-level [11, 24–26], higher socioeconomic status has been associated with better diet quality. Individuals with higher incomes and education have better diet quality [22, 27]. Higher neighbourhood socioeconomic status has been associated with diet independent of individual socioeconomic status [11, 24–26]. For example, in a recent multi-country study, the odds of fruit and vegetable intake were higher in more affluent neighbourhoods compared to less affluent neighbourhoods in several countries including Canada [11]. Findings from a Canadian study also supports the differential influence of neighbourhood socioeconomic status on diet, specifically on the consumption of high-fat, high-sugar foods [26].

The associations between neighbourhood socioeconomic status and diet may reflect the quantity and quality of available local food destinations [28, 29]. Particularly in the US, there is evidence of food deserts (i.e. low socioeconomic status urban areas that lack access to affordable, healthy foods) [30]. Compared with higher socioeconomic status neighbourhoods, lower socioeconomic status neighbourhoods have consistently been shown to have limited availability of supermarkets but greater availability of fast-food and convenience stores [31–34]. Similar, albeit less consistent observations have been made in Canada [35–37].

Though there is some evidence for the independent effects of the objectively-assessed neighbourhood food and socioeconomic environments on diet quality, evidence regarding the potential interaction effects between the neighbourhood food environment and neighbourhood socioeconomic status environment on diet are lacking [9, 19]. In the US, greater availability of convenience stores in more socioeconomically deprived neighbourhoods was associated with poorer diet quality compared to less deprived neighbourhoods [9]. Others have found no interaction between fast-food restaurant availability and neighbourhood deprivation on diet [19]. To our knowledge, no studies have examined the interactions between neighbourhood food and socioeconomic environments on diet quality in the Canadian context. Disentangling these effects is valuable for developing multi-level interventions aimed at improving diet quality; for example, informing urban development policies on ideal food destination distribution in neighbourhoods with different socioeconomic status.

The purpose of this study was to estimate the independent associations and interaction effects of the objectively assessed neighbourhood food environment and neighbourhood socioeconomic status on diet quality in Canadian adults.

## Methods

### Study design and recruitment

This study is part of a larger research project “Pathways to Health”. The study design and recruitment strategies described are those of the larger project and the data used in the current study represent a subset of participants from the main project.

A cross-sectional survey was conducted in Calgary (a large cosmopolitan area in Alberta, Canada) that included a stratified random sample of established Calgary neighbourhoods built prior to 1980 (*n* = 173). Selection of neighbourhoods built prior to 1980 helped ensure stability in the neighbourhood block patterns, which in Calgary are typically associated with different urban forms [38]. Twelve strata were defined by neighbourhood block pattern (grid, warped-grid, and curvilinear [38]) and quartiles(Q) of a neighbourhood socioeconomic status score (Q1: −5.40 to −3.53; Q2: −3.54 to 0.28; Q3: 0.29 to 2.80, and; Q4: 2.81 to 3.28). Neighbourhood socioeconomic status was estimated using a socioeconomic deprivation index derived from seven dissemination area (DA) level variables from the 2006 Canadian Census. DAs are the smallest spatial unit at which census data from Statistics Canada is available for analysis [39]. The selection of variables that reflect both social and material deprivation in Canada was informed by the work of Pampalon and colleagues [40]. The seven census variables were: proportion of 25–64 year olds whose highest education is below a high school diploma; proportion of single-parent families; proportion of rented private dwellings; proportion of divorced, separated, or widowed among those ≥15 years of age; proportion unemployed among those ≥25 years of age; median gross household income; and average value of dwellings. Dissemination area level variables [39] aggregated up to the neighbourhood boundary were standardized (i.e., converted to z-scores) to estimate area level socioeconomic status (see Additional file 1: Table S1). The 2006 Canadian Census data were used instead of the 2011 census data given methodological changes in the format of the latter Census and concerns regarding possible non-response bias [41].

One neighbourhood per stratum was selected via computer automated random sampling. The City of Calgary provided an updated database containing full household address information for all dwellings located within our study neighbourhoods. Computer automated random sampling was used to select *n* = 10,500 households from these neighbourhoods to participate in the “Pathways to Health” study.

In April 2014, a survey package was mailed to each household that included instructions for completing two self-administered online questionnaires: 1) a physical activity, health and demographic questionnaire, and 2) the Canadian Diet History Questionnaire II (C-DHQ II). A postcard introducing the study was sent one week prior, and two reminder postcards were sent two and four weeks after sending the survey package, encouraging participation. An incentive of entry into a prize draw to win a $50 voucher was also offered for participants who completed both questionnaires. One adult (≥20 years of age) per household, with the next birthday, was invited to participate in the study. Of the 10,500 households sent a survey package, 407 were non-deliverable (database address errors; vacant properties), 918 completed the online physical activity, health, and demographic questionnaire, and 480 completed the online C-DHQ II. While we cannot accurately estimate the response rate, evidence suggests that approximately only 85.7 % of Calgary households have access to the internet [42] and approximately 1.4 % of individuals in our study neighbourhoods speak neither English or French [43] and therefore ineligible to participate. Based on this information the response rate to the online C-DHQ II is approximately 5.6 %. Despite low response rates for population-based online surveys being common [44] our non-targeted recruitment strategy might explain the low response rate for our study. Nevertheless, the primary focus of this study was to estimate the magnitude and direction of associations between diet quality and neighbourhood environment characteristics in a geographically and socioeconomically diverse sample. The current study includes data only from participants who completed both online questionnaires (*n* = 446). The University of Calgary Conjoint Health Research Ethics Board approved this study (REB13-0301). A letter detailing informed consent was included in all study packages; informed consent was received for each participant in our sample.

#### Variables

##### Participants’ walksheds

**Fig. 1:**
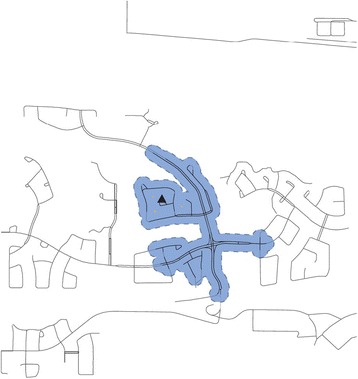
400 m walkshed created using line-based network buffer. Figure 1 Legend: Black triangle represents the location of the participant’s house. Blue, dashed-line represents the boundary of the 400 m walkshed. Solid blue region is the area included in the 400 m walkshed. Yellow triangles represent food destinations

Participants’ household addresses were geocoded. Using ArcMap (ESRI), a 400 meter (m) line-based network polygon or walkshed [45] was estimated around each participant’s home, representing the distance that could be walked in any direction within approximately five minutes (Fig. 1). A 400–450 m distance between homes and destinations has been proposed for encouraging active transportation in other countries [46, 47]. The 400 m walkshed was used to define the local neighbourhood boundary for which food and socioeconomic environments surrounding each participant’s home were estimated. Because larger walkshed areas are positively associated with greater street connectivity [48] which may be related to level of access to destinations, walkshed area was included as an overall measure of built environment in our analysis.

##### Walkshed food environment variables

**Fig. 2:**
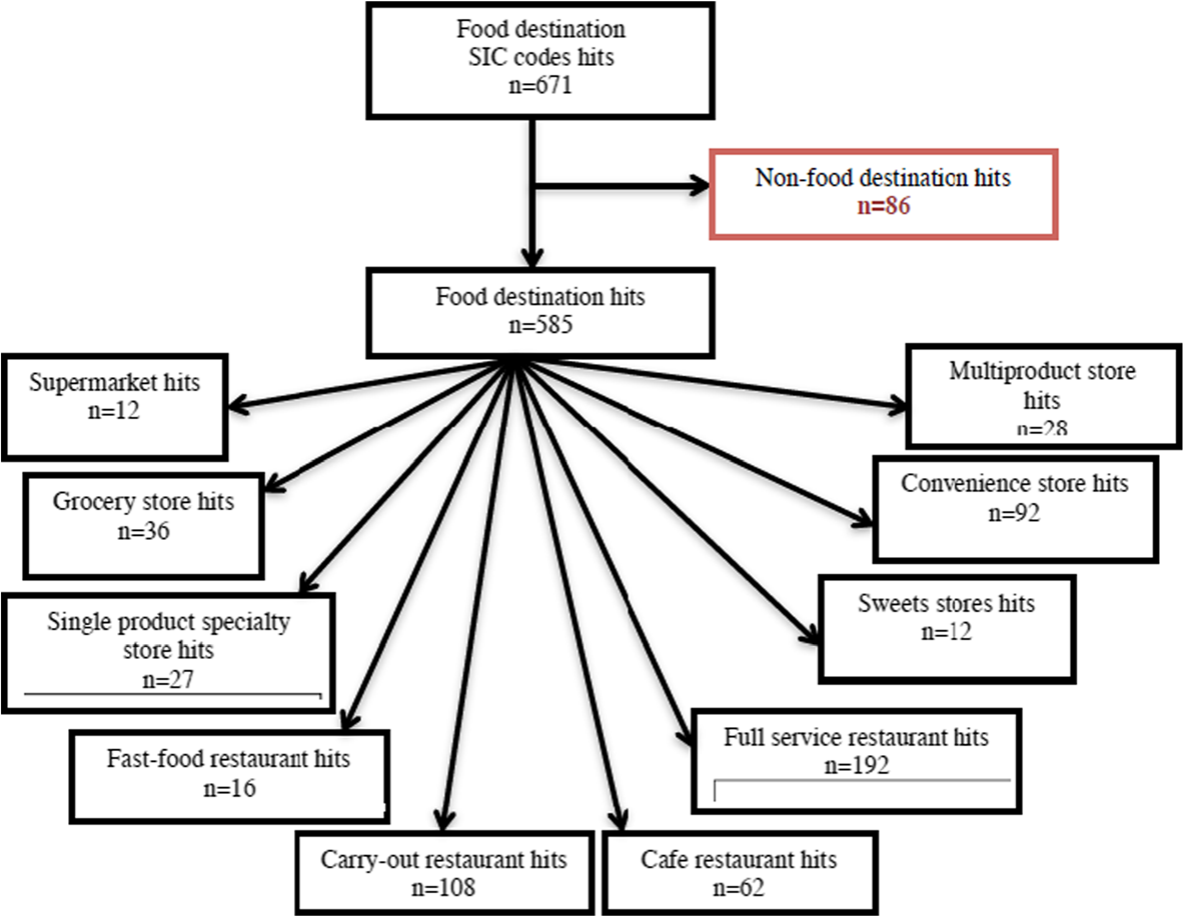
Hits* per food store destination type using ArcMap Closest Facility. Method for each 400 m walkshed. Figure 2 Legend: * Hits identify each food destination within each walkshed using the SIC codes. Hits allow one destination to be counted as many times as it appears (i.e. one food destination can be counted multiple times due to overlapping walksheds). SIC codes queried: 5399, 5411, 5421, 5431, 5441, 5451, 5461, 5499, 5541, 5812, and 5912

We included restaurants and food stores as food destinations of interest to provide a comprehensive definition of the walkshed food environment. Standard Industrial Classification (SIC) codes (5399, 5411, 5421, 5431, 5441, 5451, 5461, 5499, 5541, 5812, and 5912) and the DMTI Spatial Enhance Points of Interest file (EPOI), 2013 [49] were used to identify food destinations within the participants’ walkshed. The SIC code classification system, which assigns a four digit code to destinations based on the primary business of the establishment, was created by the United States government and was later adopted by the Government of Canada. The EPOI file is a commercial database produced by DMTI Spatial annually and receives data from variety of sources, primarily through industry partnerships [49]. The food destinations queried provided healthy and unhealthy foods and were categorized into nine food destination types [fast-food restaurants, cafés, carry-out restaurants, full-service restaurants, supermarkets, grocery stores, convenience stores, multiproduct stores selling groceries (e.g. pharmacies), and single product specialty stores (e.g.. butchers, fruit and vegetable stands, and bakeries) (Fig. 2), similar to what has been done in previous research [15, 50]. Destinations listed under some SIC codes were not relevant (e.g., SIC 5541 included gas stations with a food retail store attached as well as automobile service garages with no retail store attached), hence two research assistants independently checked the names and type of the food destinations and removed irrelevant destinations.

Similar to methods used elsewhere [15], we estimated four walkshed food environment variables: 1) presence of any food destination (whether or not there was at least one food destination within the walkshed); 2) density of food destinations (total count of food destinations within the walkshed); 3) diversity of food destinations (the count of different food destinations types [minimum = 0, maximum = 9] within the walkshed), and; 4) presence of any key food destination types (key food destination types were selected based on the Retail Food Environment Index (RFEI) [51]. Key food destination categories were: supermarket or grocery store; convenience or multiproduct store; and any restaurant. We did not make the distinction between “healthy” and “unhealthy” food destinations because our outcome was focused on diet quality (which captures nutrient ‘variety’, ‘moderation’, and ‘adequacy’), and whether a food destination offers solely or mostly “healthy” versus unhealthy” food is difficult to distinguish, especially based on spatial data only. Presence was defined as whether or not there was at least one destination from each of the key categories within the walkshed. Given that the presence and count of food destinations are dependent on walkshed size area, which can differ among participants when using a network-based estimation approach [48], we normalized all food environment variables by dividing by walkshed geographical area (km^2^).

##### Walkshed socioeconomic status

**Fig. 3:**
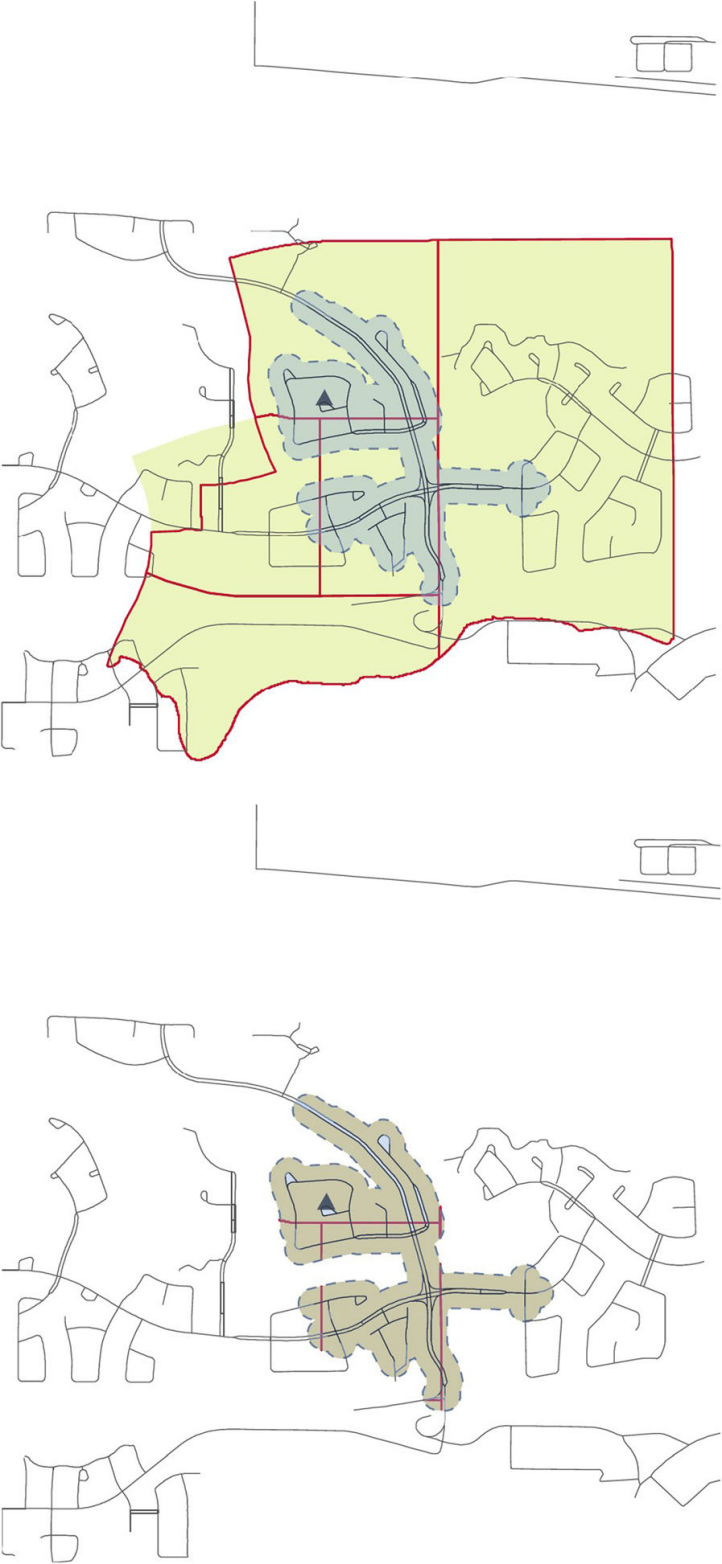
**a** Areal interpolation - polygon containment method. **a** Legend: Black triangle represents the location of the participant’s house. Blue, dashed-line represents the boundary of the 400 m walkshed. Solid blue region is the area included in the 400 m walkshed. Solid red lines represent Canadian Census dissemination area boundaries. Yellow-beige region represents the area of the socio-demographic population characteristics that would be included for estimating socioeconomic status if the polygon containment method was used. **b** Areal interpolation - buffer containment method. **b** Legend: Black triangle represents the location of the participant’s house. Blue, dashed-line represents the boundary of the 400 m walkshed. Solid red lines represent Canadian Census dissemination area boundaries. Beige region represents both the area included in the 400 m walkshed and the area of the socio-demographic population characteristics that were included when estimating socioeconomic status using the buffer containment method

Dissemination area (DA) level Census data were used to estimate the area-weighted, seven variable socioeconomic deprivation index for each walkshed using areal interpolation and the buffer containment method [52]. The buffer containment method provides accurate estimates of walkshed socioeconomic status because it counts only population characteristics that fall within the boundaries of the walkshed polygon and weights the estimates according to the amount of overlap between the walkshed and DA boundary (i.e. walksheds that intersect multiple DAs are modeled, see Fig. 3a, b) [53]. Higher index values represented higher walkshed deprivation.

##### Diet quality

Participants completed the online Canadian Diet History Questionnaire II (C-DHQ II) [54] a 165-item past-year food frequency questionnaire that was adapted from the US Diet History Questionnaire II (DHQ II) for the Canadian population [55]. The C-DHQ II food list and accompanying nutrient database were created using the Canadian Community Health Survey (CCHS 2.2, Nutrition) data [56] and reflects food consumed and available in Canada. The C-DHQ II asks respondents to estimate the usual frequency and portion size of foods consumed in the past 12 months [54, 55]. The C-DHQ II has not yet been validated. However, given its similarities to the US DHQ I which has been validated, it is thought to have moderate ability to capture diet with a significant underestimation of energy and protein intake [54]. The C-DHQ II nutrient database and accompanying Diet*Calc software were used to analyze the C-DHQ II questionnaire responses.

Eight new variables representing Canada’s Food Guide (CFG) serving sizes were created for each food-based item on the C-DHQ II and added to the existing nutrient database. These variables were required for the estimation of the Canadian Health Eating Index (C-HEI) [22] and are the CFG serving size equivalents for: total fruit; vegetables; whole fruit; dark green and orange vegetables; total grain; whole grains; milk and alternative; meats and alternatives; and the caloric value of “other foods” (as described in the CFG [57]). A similar method for creating serving size equivalents is described elsewhere [58].

Participant C-DHQ II responses were analyzed using the C-HEI variables and Diet*Calc (Version 1.5.0), a freely available software program that uses a data dictionary modified for the C-DHQ II. CFG serving size intakes provided in the output from Diet*Calc for each participant were then further analyzed to estimate a C-HEI score [22] as a measure of diet quality. The C-HEI has high content and construct validity and is appropriate for use in the Canadian population [19]. The C-HEI score can range from 0 (poor quality) to 100 (high quality) reflecting total diet quality based on criteria described in Table 1. Adequacy components capture nutrient and food intake sufficiency and include the total consumption of fruits and vegetables, whole fruit, dark green and orange vegetables, milk and alternatives, meat and alternatives, and, polyunsaturated fatty acids [22]. The moderation components capture excessive intake of nutrients and foods and include: sodium, saturated fatty acids, and other foods not recommended in the CFG [22]. Higher C-HEI scores represent closer adherence to Canadian dietary recommendations [22, 57].

**Table 1:** Scoring criteria for Canadian adapted Healthy Eating Index (C-HEI)^a^

COMPONENT	RANGE OF SCORES	SCORING CRITERIA
Adequacy^c^	0 to 60 points	
Total vegetables and fruit	0 to 10 points	Minimum: 0
		Maximum: 4 to 10 servings^b^
Whole fruit	0 to 5 points	Minimum: 0
		Maximum: 0.8 to 2.1 servings (21 % of recommendation for total vegetables and fruit)^b^
Dark green and orange vegetables	0 to 5 points	Minimum: 0
		Maximum: 0.8 to 2.1 servings (21 % of recommendation for total vegetables and fruit)^b^
Total grain products	0 to 5 points	Minimum: 0
		Maximum: 1.5 to 4 servings (50 % of recommendation for total grain products)^b^
Milk and alternatives	0 to 10 points	Minimum: 0
		Maximum: 2 to 4 servings^b^
Meat and alternatives	0 to 10 points	Minimum: 0
		Maximum: 1 to 3 servings (75 to 225 g)^b^
Unsaturated fats	0 to 10 points	Minimum: 0
		Maximum: 30 to 45grams^b^
Moderation^d^	0 to 40 points	
Saturated fats	8 to 10 points	Maximum 7 % to 10 % of total energy intake
	0 to 8 points	
Sodium	8 to 10 points	Adequate intake to tolerable upper intake level
	0 to 8 points	
“Other food”	0 to 20 points	Minimum: 5 % or less of total energy intake Maximum: 40 % or total energy intake

^a^Garriguet, D., 2009. Diet quality in Canada. Heal. Reports 20, 41–52 [22]

^b^according to age and sex, as specified in *Canada’s Food Guide*

^c^for adequacy components, 0 points for minimum or less, 5 or 10 maximum or more, and proportional for amounts between minimum and maximum

^d^for moderation components, 10 or 20 points for minimum or less, 0 points for maximum or more, and proportionally between minimum and maximum

##### Socio-demographic and health-related characteristics (covariates)

Diet quality has many correlates [5, 22, 59]. Covariates associated with diet quality and transport within the neighbourhood in previous literature [2, 5, 22, 27, 59–61] were captured from the online physical activity, health and demographic questionnaire and included: sex; age (20–39 years, 40–59 years or ≥60 years); race (Caucasian or other races); marital status (married or living with partner, or, other arrangement); dependents (any age) at home (no dependent, or, at least 1 dependent); highest education completed (high school or less, college/diploma/trade, or university); gross household income (≤$59 999, $60 000–$119 999, ≥$120 000, or refused to answer); smoking status (daily or occasional smoker, or, non-smoker); car available for personal use (always, or never/sometimes); dog ownership (owner or non-owner); self-reported mental and physical health (poor, fair, good, very good, or excellent); hours spent sitting per day, and hours spent in the neighbourhood during a typical week.

#### Statistical analyses

Descriptive statistics including means (and standard deviations) and frequencies were estimated for the socio-demographic, health-related, and neighbourhood environment characteristics of the sample. Pearson correlations were used to estimate bivariate linear associations between the walkshed socioeconomic deprivation index, walkshed area, walkshed food destination density, food destination diversity, and C-HEI scores. C-HEI scores between socio-demographic groups were compared in bivariate analyses based on a linear regression models. Multivariable linear regression (unstandardized β and 95 % CI) was used to regress C-HEI scores on the walkshed socioeconomic deprivation index and walkshed food environment variables (presence, density, diversity, and key destination types), while adjusting for walkshed area and participant reported socio-demographic and health-related characteristics. Interaction terms between the walkshed socioeconomic deprivation index and each walkshed food environment variable (presence, density, diversity, and key destination types) were tested within the fully-adjusted main effects model using backward stepwise selection. Interaction terms with a *p* > 0.10 were removed from the final models. We estimated variance inflation factors (VIF) for each model to check for multicollinearity.

## Results

### Sample characteristics

After removing participants with incomplete data required for regression analysis (*n* = 34), the final analytic sample consisted of 466 participants. The sample consisted of 61.4 % women, 79.6 % aged ≥40 years, 93.5 % were Caucasian, 78.5 % were married or living with a partner, 70.0 % were university educated, 77.2 % had a gross household income > $60 000, 96.4 % were non-smokers, 91.3 % had access to a vehicle for personal use, 73.8 % reported at least very good mental health, 61.4 % reported at least very good health physical (Table 2). Compared with population based census-derived estimates for our study neighbourhoods, our sample was older and had underrepresentation from men, non-Caucasians, those not married or common law, and those least educated (Table 2). The mean (standard deviation, SD) walkshed area was 0.19 km^2^ (0.05). Walkshed socioeconomic deprivation index scores ranged from −7.2 to 5.3 while the mean (SD) was −2.3 (1.9), indicating low deprivation (higher socioeconomic status).

**Table 2:** Socio-demographic and environment characteristics (*n* = 446)

Characteristic	Total	Population estimates^b^
Participants [n]	446	
Sex [%]		
Men	38.6	49.7
Women	61.4	50.3
Age [%]		
21–39 years	20.4	38.9
40–59 years	44.8	38.9
≥ 60 years	34.8	22.1
Race [%]		
Caucasian	93.5	
All other races	6.5	22.4 (visible minority)
Marital status [%]		
Married/living with partner	78.5	55.6
Others	21.5	44.4
Dependents at home (all ages) [%]		
0 dependents	52.2	
≥ 1 dependent	47.8	0.77/household (median)
Highest education completed [%]		
High school or less	12.6	36.6
College/trade/diploma	17.5	
University	70.0	63.4
Gross annual household income [%]		
≤ $59 999	8.7	
$60 000–$119 999	33.9	$85,478 (median)
$ ≥ 120 000	43.3	
Refused/don’t know	14.1	
Smoking status in past 12 months [%]		
Daily or occasional	3.6	
Non-smoker	96.4	
Car available for personal use [%]		
Always	91.3	
Never or sometimes	8.7	
Dog ownership in past 12 months [%]		
Owner	35.2	
Non-owner	64.8	
Self-reported mental health [%]		
Poor/fair/good	26.2	
Very Good	44.2	
Excellent	29.6	
Self-reported physical health [%]		
Poor/fair/good	38.6	
Very Good	41.0	
Excellent	20.4	
Sitting hours/day [mean (SD)]	7.3 (4.0)	
Hours in neighbourhood/typical week [mean (SD)]	109.0 (31.9)	
Walkshed level socioeconomic deprivation index^a^ [mean (SD)]	−2.3 (2.9)	
Walkshed area (km^2^) [mean (SD)]	0.19 (0.05)	

^a^Walkshed level socioeconomic deprivation (Socioeconomic Disadvantage) index includes: percent of 25 to 64 year olds with no diploma certificate or degree; percent of lone parent families among all census families; percent of private dwellings rented; percent of divorced or separated or widowed 15+ years; unemployment rate for those 25+ years; median gross income; and average value of the dwelling). Street network level socioeconomic deprivation calculated for each 400 m line-based around participants geo-coded address. Higher index scores represent higher socioeconomic disadvantage

^b^City of Calgary 2014 Community Profiles data. These estimates are based on data from the 2011 Canadian Census and the 2014 Calgary Civic Census for the 12 study neighbourhoods. All estaimtes were averaged across the 12 study neighbourhoods

Over one quarter (27.1 %) of walksheds had at least one food destination. The mean walkshed (SD) density of food destinations/km^2^ was 6.0 (17.4)/km^2^ while diversity was 3.6 (7.5) stores/km^2^. For key food destinations types, 13.2 % of walksheds had at least one supermarket or grocery store, 15.7 % had at least one convenience or multiproduct store, and 22.9 % had at least one restaurant.

The mean C-HEI score for the sample was 66.4 (10.8). In the bivariate analyses based on linear regression models, women had statistically significantly higher C-HEI scores compared to men [66.3 (95 % confidence interval 65.0–67.5) versus 61.5 (CI 59.9–63.1)] and non-smokers had statistically higher C-HEI scores compared to daily or occasional smokers [64.7 (CI 63.7–65.7) versus 56.9 (CI 50.6, 63.2)]. There was no statistically significant difference in C-HEI scores by any other socio-demographic or health-related characteristics (results not shown in a table).

### Associations between walkshed food destinations, socioeconomic deprivation index, socio-demographic/ health-related characteristics, and diet quality

We found modest but statistically significant (*p* < .05) correlations between walkshed socioeconomic deprivation index and food destination variables. Greater walkshed deprivation was significantly correlated with higher food destination density (*r* = 0.23) and higher food destination diversity (*r* = 0.31). Walkshed area (km^2^) was also significantly correlated with food destination density (*r* = 0.15) and food destination diversity (*r* = 0.17). Food destination density and diversity were highly correlated (*r* = 0.87). There were no statistically significant correlations between walkshed food destination variables or socioeconomic deprivation index and C-HEI scores.

The bivariate linear regression estimates suggested no associations between the C-HEI and any walkshed food destination variables or walkshed socioeconomic deprivation (Table 3). Similarly, after adjusting for socio-demographic, health-related characteristics and built environment there was no evidence of statistical interactions between walkshed socioeconomic deprivation and any walkshed food destination variables in any of the multivariable main effects regression models. However, in the main effect model for food destination density, for every one food destination/km^2^ increase within the walkshed C-HEI score increased by 0.06 points (95 % CI 0.01–0.12). Although statistically significant, the magnitude is small. Presence, diversity, and key food destinations types within the walkshed were not statistically associated with C-HEI scores in main effects model. Across all models multicollinearity was not considered a problem (VIF: minimum = 1.0 and maximum = 4.6).

**Table 3:** Unadjusted and adjusted estimates of the associations between walkshed food environment characteristics, covariates and diet quality (C-HEI score)

	Unadjusted^+^	Model 1	Model 2	Model 3	Model 4	Model 5	Model 6
		Food destination presence ^a^	Food destination density^b^	Food destination diversity^c^	Supermarket/ grocery store presence^d^	Convenience/multi-product store presence^e^	Restaurant presence^f^
	β (95 % CI)	β (95 % CI)	β (95 % CI)	β (95 % CI)	β (95 % CI)	β (95 % CI)	β (95 % CI)
Intercept (β_0_)		58.15 (50.75, 65.54)	57.56 (50.22, 64.90)	57.58 (50.20, 64.96)	57.49 (50.08, 64.91)	57.67 (50.27, 65.05)	57.90 (50.51, 65.29)
Unadjusted^+^ β (95 % CI)		−0.98 (−3.23, 1.26)	0.05 (−0.01, 0.10)	0.04 (−0.09, 0.17)	−1.05 (−4.72, 2.63)	0.35 (−2.40, 3.10)	−0.62 (3.00, 7.80)
Adjusted^~^ β (95 % CI)		−1.32 (−3.58, 0.94)	0.06 (0.01, 0.12)*	0.06 (−0.08, 0.19)	−0.86 (−4.50, 2.77)	0.44 (−2.37, 3.25)	−0.71 (−3.11, 1.68)
Covariates							
Sex^^^							
Women	4.77 (2.76, 6.78)^#^	4.65 (2.59, 6.71)*	4.84 (2.78, 6.90)*	4.73 (2.66, 6.79)*	4.65 (2.59, 6.71)*	4.69 (2.63, 6.78)*	4.66 (2.60, 6.72)*
Age^^^							
40–59 years	−1.35 (−4.03, 1.32)	−1.66 (−4.28, 0.96)	−1.32 (−3.91, 1.27)	−1.34 (−3,95, 1.27)	−1.46 (−4.07, 1.32)	−1.37 (−4.06, 1.25)	−1.54 (−4.15, 1.07)
≥ 60 years	−1.49 (−4.28, 1.30)	−0.37 (−3.60, 2.85)	−0.26 (−3.46, 2.94)	−0.11 (−3.32, 3.10)	−0.13 (−3.34, 3.09)	−0.11 (−3.35, 3.14)	−0.26 (−3.48, 2.96)
Race^^^							
All other races	1.05 (−3.01, 5.11)	2.96 (−1.04, 6.96)	3.30 (−0.68, 7.23)	3.26 (−0.74, 7.26)	3.09 (−0.91, 7.08)	3.19 (−0.81,7.19)	3.05 (−7.06, 0.95)
Marital status^^^							
Others	−3.00 (−5.41, −0.57)	−1.34 (−3.90, 1.21)	−1.50 (−4.06, 1.05)	−1.41 (−3.97, 1.16)	−1.23 (−3.80, 1.35)	−1.34 (−3.90,1.22)	−1.31 (−3.89, 1.25)
Dependents at home^^^							
≥ 1 dependent	2.29 (0.30, 4.28)	1.90 (−0.27, 4.07)	2.11 (−0.06, 4.27)	2.03 (−0.14, 4.21)	1.99 (−0.18, 4.16)	2.00 (−0.18, 4.20)	1.93 (−0.24, 4.11)
Highest education completed^^^							
College/trade/diploma	3.38 (−0.30, 7.05)	1.81 (0.03, 7.15)*	3.31 (−0.24, 6.85)	3.39 (−0.17, 6.98)	3.50 (−0.07, 7.08)	3.47 (−0.09, 7.03)	3.55 (−0.01, 7.11)
University	4.29 (1.24, 7.34)	1.58 (0.16, 6.36)*	2.69 (−0.41, 5.80)	2.89 (−0.23, 6.01)	3.14 (0.04, 6.24)*	3.01 (−0.09, 6.12)	3.19 (0.08, 6.31)*
Total gross household income^^^							
$60 000–$119 999	0.42 (−3.36, 4.21)	−0.36 (−4.14, 3.42)	−0.50 (−4.26, 3.28)	−0.37 (−4.15, 3.40)	−0.26 (−4.05, 3.53)	−0.37 (−4.16, 3.42)	−0.34 (−0.41, 3.44)
$ ≥ 120 000	2.63 (−1.07, 6.33)	1.26 (−2.74, 3.42)	1.02 (−2.98, 5.01)	1.18 (−2.83, 5.19)	1.47 (−2.56, 5.51)	1.22 (−2.79, 5.23)	1.27 (−2.73, 5.28)
Refused	0.44 (−3.91, 4.73)	−1.07 (−2.73, 5.27)	−1.75 (−6.04, 2.56)	−1.36 (−5.65, 2.94)	−0.97 (−5.28, 3.33)	−1.25 (−5.54, 3.04)	−1.16 (−5.45, 3.12)
Smoking status in past 12 months^^^							
Non-smoker	7.86 (2.52,13.20)^#^	8.90 (3.76, 14.04)*	9.14 (4.02, 14.26)*	9.10 (3.96, 14.23)*	9.15 (4.00, 14.29)*	9.14 (3.98, 14.31)*	9.02 (3.89, 14.21)
Car available for personal use^^^							
Never or sometimes	−0.45 (−4.00, 3.10)	−1.60 (−5.01, 1.82)	−1.27 (−4.68, 2.15)	−1.42 (−4.84, 1.99)	−1.46 (−4.87, 1.96)	1.47 (1.96, 4.87)	1.50 (−1.91, 4.91)
Dog ownership in past 12 months^^^							
Non-owner	−1.70 (−3.80, 0.40)	−2.45 (−4.50,-0.41)*	−2.25 (−4.29, −0.20)*	−2.36 (−4.40, −0.32)*	−2.47 (−4.52 -0.43)*	−2.41 (−4.45, −0.36)	−2.41 (−4.45,-0.36)*
Self-reported mental health^^^							
Very Good	−1.17 (−3.64, 1.29)	−3.50 (−6.04, −0.95)*	−3.15 (−5.67, −0.62)*	−3.20 (−5.73, −0.66)*	−3.24 (−5.78, −0.70)*	−3.25 (−5.80, −0.69)*	−3.37 (−5.91, 0.83)*
Excellent	0.76 (−1.92, 3.43)	−1.18 (−4.17, 1.80)	−0.91 (−3.87, 2.06)	−0.97 (−3.95, 2.01)	−0.86 (−3.86, 2.14)	−0.99 (−4.00, 2.00)	−1.08 (−4.07, 1.89)
Self-reported physical health^^^							
Very Good	3.62 (1.39, 5.83)	3.59 (1.24, 5.93)*	3.44 (1.10, 5.78)*	3.52 (1.17, 5.86)*	3.44 (1.10, 5.80)*	3.52 (1.17, 5.86)*	3.54 (1.20, 5.89)*
Excellent	3.51 (0.80, 6.22)	2.23 (−0.88, 5.34)	1.90 (−1.20, 5.00)	2.07 (−1.03, 5.19)	2.14 (−0.97, 5.24)	2.12 (−0.98, 5.24)	2.21 (−0.90, 5.33)
Sitting hours/day	−0.32 (−0.57, −0.68)	−0.14 (−0.41, 0.12)	−0.13 (−0.39, 0.14)	−0.14 (−0.40, 0.13)	−0.14 (−0.40, 0.12)	−0.13 (−0.40, 0.13)	−1.08 (−4.07, 1.89)
Hours in neighbourhood/ typical week	0.05 (0.02, 0.08)^#^	0.06 (0.03, 0.09)*	0.06 (0.02, 0.09)*	0.06 (0.02, 0.09)*	0.06 (0.02, 0.09)*	0.06 (0.02, 0.09)*	0.06 (0.02, 0.09)*
Walkshed-level socioeconomic deprivation Index^Ŧ^	−0.24 (−0.59, 0.11)	−0.08 (−0.46, 0.30)	−0.24 (−0.61, 0.14)	−0.19 (−0.57, 0.19)	−0.15 (−0.52, 0.23)	−0.16 (−0.53, 0.22)	−0.11 (−0.48, 0.27)
R^2^		0.18	0.19	0.18	0.18	0.18	0.18

^+^Unadjusted estimates for the association between walkshed food environment measure (presence, density, diversity, presence of supermarket/grocery store, presence of convenience/multiproduct store, presence of restaurant) and C-HEI score are presented in the row with the “unadjusted” heading. Unadjusted estimates of the association between each covariate and C-HEI scores are presented in the first column of the table

^~^Adjusted estimates control for all covariates (sex, age, race, marital status, dependents at home, level of education, total gross household income, smoking status, car availability for personal use, dog ownership, self-reported physical health, self-reported mental health, number of hours spent sitting per day, number of hours spent in the neighbourhood during a typical week and walkshed level socioeconomic deprivation). These covariates represent socio-demographic, socioeconomic, health behaviours, and neighbourhood characteristics previously noted to be associated with diet quality. Fully-adjusted estimates are intended to isolate the effects of the walkshed socioeconomic status and walkshed food environment on diet quality

^a^Model 1 used linear regression to estimate the association between the presence of any food destination within the 400 m walkshed and C-HEI score. Presence was defined as at least one food destination present within the 400 m walkshed. The intention of this model was to determine if having a food destination within a 400 m walkshed of home address, regardless of type or count, was associated with diet quality (C-HEI score). All covariate estimates in the Model 1 column are fully-adjusted

^b^Model 2 used linear regression to estimate the associations between the density of food destinations within the 400 m walkshed and C-HEI score. Density was a continuous variable and defined as the total count of food destinations (all types) per walkshed area (km^2^). The intention of this model was to determine if the number of food destinations, regardless of type, within a 400 m walkshed of home address was associated with diet quality (C-HEI score). All covariate estimates in the Model 2 column are fully-adjusted

^c^Model 3 used linear regression to estimate the associations between the diversity of food destinations within the 400 m walkshed and C-HEI score. Diversity was defined by an index variable [minimum = 0, maximum = 9] capturing the variety of food destination types available within 400 m from home address. The nine food destination types were: fast-food restaurants, cafés, carry-out restaurants, full-service restaurants, supermarkets, grocery stores, convenience stores, multiproduct stores selling groceries (e.g. pharmacies), and single product specialty stores (e.g.. butchers, fruit and vegetable stands, and bakeries). Presence of a food destination type was defined as at least one destination within the 400 m walkshed. The intention of this model was to determine if greater diversity in food purchase opportunity within a 400 m walkshed of home address was associated with diet quality (C-HEI score). All covariate estimates in the Model 3 column are fully-adjusted

^d^ Model 4 used linear regression to estimate the associations between the presence of a supermarket or grocery store within the 400 m walkshed and C-HEI score. Presence was defined as at least one supermarket or grocery store within the 400 m walkshed. The intention of this model was to determine if the presence of a food destination assumed to offer opportunity to purchase a variety of food types (e.g., fresh produce, lean proteins, dairy, whole grains) was associated with diet quality (C-HEI score). All covariate estimates in the Model 4 column are fully-adjusted

^e^Model 5 used linear regression to estimate the associations between the presence of a convenience or multiproduct store within the 400 m walkshed and C-HEI score. Presence was defined as at least one convenience or multiproduct store within the 400 m walkshed. The intention of this model was to determine if the presence of a food destination assumed to offer opportunity for limited variety of food purchase (e.g., primarily packaged and high fat, high sugar convenience foods) was associated with diet quality (C-HEI score). All covariate estimates in the Model 5 column are fully-adjusted

^f^Model 6 used linear regression to estimate the associations between the presence of a restaurant within the 400 m walkshed and C-HEI score. Presence was defined as at least one restaurant within the 400 m walkshed. The intention of this model was to determine if the presence of a restaurant assumed to offer limited variety of opportunity for food purchase (e.g., prepared dishes often high in sodium and fat) was associated with diet quality (C-HEI score). All covariate estimates in the Model 6 column are fully-adjusted

^Reference groups: Model1 = 0 food destinations within 400 m street network; Model 4 = 0 supermarket or grocery stores within 400 m street network, 0 convenience or multiproduct stores within 400 m street network, 0 restaurants within 400 m street network; Sex = men; Age = 21–39 years; Race = Caucasian; Marital status = married or living with partner; Dependents at home = no dependents at home; Highest education = high school diploma or less; Total gross income = ≤$59 000; Smoking status = non-smoker; Car available for personal use = always have a car available for personal use; Dog ownership = owner; Self-reported mental health = poor/fair/good; Self-reported physical health = poor/fair/good

*Statistically significant at alpha = 0.05

^#^Statistically significant at alpha = 0.003 (Bonferonni adjustment)

In all multivariable main effects regression models, self-reported mental health was statistically significantly associated with C-HEI score with those reporting very good mental health having lower C-HEI scores than those reporting poor or fair mental health (Table 3). Conversely, better self-reported physical health, dog ownership, and spending more time in the neighbourhood were positively and statistically significantly associated with C-HEI scores. No other socio-demographic and health-related covariates were statistically significantly associated with C-HEI scores in the multivariable main effects regression models.

## Discussion

We found a higher the number of food destinations within 400 m of home, regardless of food destination type, is associated with higher diet quality scores in Canadian adults, even after accounting for walkshed level socioeconomic status and other individual-level characteristics. No other walkshed food destination variables were associated with diet quality, which is in contrast to US and European evidence hat found specifically, the presence and density of supermarkets within the neighbourhood to be associated with higher diet quality [16, 17] while presence of convenience stores and density of fast-food restaurants is associated with lower diet quality [9, 62]. Further, our findings did not support previous research suggesting a relation between neighbourhood socioeconomic status and diet [11, 25]. Notably, we found no significant interactions between the walkshed food destination variables and walkshed socioeconomic status in relation to diet quality. This finding is similar to Boone-Heinonen et al. [19] who found no interaction between fast-food restaurant availability and neighbourhood deprivation on diet. In addition, we found that diet quality was higher with greater time spent in the neighbourhood, in study participants who reported very good physical health, as well as those reporting poor/fair/good mental health, those who were dog owners, non-smokers, and among women.

In contrast to findings in the US where increased density of supermarkets within the neighbourhood is associated with better diet quality [17, 18], and, a higher density of fast-food restaurants and convenience stores is associated with poorer diet quality [19, 62], the presence of supermarkets or grocery stores, convenience or multiproduct stores, and restaurants within 400 m of participants’ homes was not associated with diet quality in our study. Lack of relation between key food destinations types within 400 m walkshed and diet quality may partially be explained by the high proportion of participants who reported always having access to a car, which could allow them to access food destinations outside of their immediate walksheds. Our null finding between key food destinations types and diet quality is consistent with other Canadian findings [15] where no relation between presence of similar food destination types and diet quality within the neighbourhood (defined as a 1 km street based network) was found.

Our findings show that a greater density of food destinations (all types) within the local neighbourhood (400 m walkshed) has a weak, but positive, association with diet quality. Notably, only 27 % of our participants had at least one food destination within their walkshed while only 10 % had 20 or more food destinations/km^2^. A benefit of living in urban centers is that higher population density yields more amenities which can thus improve consumers’ access to a variety of goods and services (a concept referred to as “gains from variety”) [20, 21]. In our study, food destination density (count of food destinations) and diversity (the number of different food destination types) were statistically significantly correlated providing evidence of this relation in our study. Higher food destinations density, therefore, may increase the variety of foods available, decrease food prices (through competitive market), and allow for shorter trips to access food destinations [21]. In turn, this greater access may provide residents of neighbourhoods with higher food destination density more opportunity to make dietary choices that contribute positively to diet quality while reducing some transport and economic related barriers.

We did not find that walkshed socioeconomic status, independently or in conjunction with walkshed food destination variables, was significantly associated with diet quality. Previous studies suggest lower neighbourhood socioeconomic status is independently associated with poorer dietary habits and that this finding is potentially a function of disparities in neighbourhood food environment [32, 63, 64]. Such findings support the debated notions of food deserts [30, 65] and deprivation amplification (more deprived neighbourhoods are more likely to lack health promoting resources, and to be exposed to more health damaging resources) [66, 67]. However, we found that lower walkshed socioeconomic status was significantly correlated with greater food destination density and diversity, a result that is similar what has been observed in Montreal [37] and the UK [10], where the most deprived areas often have the best access to food destinations. Given we also found no statistically significant difference in diet quality by walkshed socioeconomic status, our study may suggest that in Calgary, Canada differences in diet quality may not be attributable to deprivation amplification in the form of food deserts. Therefore, our study suggests that, unlike the US, diet quality in Canada may not be dependent on neighbourhood socioeconomic status and that potential interventions to address diet quality can be applied across all neighbourhoods, regardless of socioeconomic status. An important limitation to acknowledge however is that our sample was of higher socioeconomic status despite our effort to capture representation from across the socioeconomic spectrum using a stratified sample design. As result, there may not have been sufficient variation in socioeconomic status at the walkshed level to detect an association between socioeconomic status and diet quality or an interaction between walkshed socioeconomic status and food environment on diet quality.

Although not the primary objective of our study, the relations we found between key covariates and diet quality were consistent with existing evidence. For example, as has been found in Canadian and American studies elsewhere [22, 27], we found women had significantly better diet quality than men. We also found non-smokers had better diet quality compared to smokers which has also previously been observed in the Canadian population [22]. Other studies have found that increasing age, individual income, and education levels are positively associated with diet quality [22]. This trend may be attributable to a parallel increase in health consciousness and nutrition knowledge with age [68]. In our study, these associations were consistent in direction with existing evidence though not always statistically significant.

Unlike many studies, we accounted for self-perceived health (physical and mental) in our multivariable regression models as a proxy for general health status given that the presence or absence of diseases, conditions, or treatments/medications can impact dietary behaviours [2, 5, 69] and potentially access to destinations. Reporting very good physical health was associated with over a 3 point higher diet quality score compared with poor/fair/good self-reported physical health. This association is plausible given that those who perceive themselves as physically healthy may do so because they are proactive in health enhancing behaviours [70] and have physiological function to support consuming a high quality diet. Interestingly, those who reported poor/fair/good mental health had significantly better diet quality compared with those who reported very good or excellent mental health. This association is contrary to what is expected given poor mental health has been associated with lower diet quality [2]. This reverse observation may be because those who have poorer mental health spend more time at home and may have received mental health counseling that included other aspects of well-being such as diet. This unique finding warrants further exploration in future studies. Another novel finding was the relation between dog ownership and better diet quality. Current evidence supports associations between dog ownership and increased physical activity [61] and better cardiovascular outcomes [71] compared with non-owners, however, to our knowledge there is no evidence to date on the association between dog ownership and dietary outcomes. The association between dog ownership and diet quality within the context of chronic disease risk reduction and management is one that warrants further exploration given that the way in which owners of pets with diabetes interact, interpret, and respond to the diagnosis and management of their pet’s diabetes may influence their interpretation and response to preventing and managing human (illnesses) diabetes [59]. Finally, time spent in the neighbourhood was used as a proxy for exposure to the walkshed food destinations. Increasing time in the neighbourhood was positively associated with diet quality. This association may exist because those who stay in the neighbourhood may tend to cook and eat at home rather than leaving the neighbourhood to eat at restaurants. Furthermore, home-cooked meals tend to be associated with higher quality food consumption [60, 72]. The abovementioned differences in diet quality observed by socio-demographic and health-related characteristics demonstrate that changes to the local food environment may be an important factor in diet quality but are not sufficient to make population level changes and re-iterate the importance of implementing dietary interventions that address factors across multiple levels of influence [73].

### Strengths and limitations

Despite the majority (85.7 %) of Calgary households reporting internet access [42], online administration of questionnaires may have excluded some individuals from participating in the study, and in particular those with the lowest socioeconomic status. Further, the low response rate limits the generalizability of the results to the broader population; however, the findings are useful for informing future hypotheses. From our cross-sectional data, we are unable to infer causality and reliance on self-report data means there is the potential for reporting bias and memory errors. While convenient, the EPOI DTMI Spatial database used to identify food destinations also has some limitations. Montréal researchers found approximately a 77 % reliability (agreement between the database and ground audit for destination existence, name, and location) [74]. Further, the EPOI database available did not have the desired and highly informative codes (the North American Industrial Classification System [NAICS]) which meant older, less specific SIC codes were used. Hence, SIC codes required manual categorization of food destinations.

A strength of our study is the use of GIS line-based networks to define the participants’ local neighbourhood (400 m walksheds) since they provide a more relational view of interaction with space [75] and allow for the fact that people often cross administratively defined neighbourhood boundaries to access shops and services [76]. A limitation however, is that our findings for the 400 m walkshed may not be replicable for larger or even smaller walkshed sizes. The use of the smaller walkshed boundary means that food destinations within walking distance to home were assessed which means limited access to a motor vehicle may be less of a barrier to accessing these destinations. Furthermore, the use of a larger walkshed size would have resulted in less variation in the food destination variables because of the increased overlap in shared environments resulting from participants being recruited from the same 12 administrative neighbourhoods.

Similar to other studies in this research area, we assumed that where people live is where they shop and eat. There are several limitations with this assumption given there is some evidence showing that shopping and eating often occur outside of the neighbourhood [77, 78]. Furthermore, we did not have any information on participants’ food shopping behaviour given that this data collection was outside the scope of our study design. Notably, we included a measure of time spent in the neighbourhood as a covariate, used to adjust for exposure to the walkshed food environment [60]. Further, we did not examine how participants travelled to food destinations; however, this should be examined in future studies. Finally, while previous studies [62] have only considered one or two food destination types, our study provided a more complete assessment of the associations between the neighbourhood food destinations and diet quality [28] as we considered nine food destination types in our study.

## Conclusions

Our findings suggest that higher food destination density within the local neighbourhood might be positively associated with diet quality among Canadian adults. This finding was independent of socio-demographic and health-related characteristics and the socioeconomic status of the local neighbourhood. With support for future studies, these findings may help inform urban planning and policies concerning food destination placement and zoning so that neighbourhoods can better support procurement and consumption of a high quality diet.

## Additional file

Additional file 1: Table S1. Principle Component Analysis results, and inter-item correlation estimates, for administrative boundary level census-derived socioeconomic status. (PDF 225 kb)
