# Supplementary material for: Associations between the neighbourhood food environment, neighbourhood socioeconomic status, and diet quality: An observational study
Source: BMC Public Health. 2016 Sep 15;16:984. doi: 10.1186/s12889-016-3631-7 (PMC5025628; doi:10.1186/s12889-016-3631-7)
Supplement: Additional file 1: Table S1. — Principle Component Analysis results, and inter-item correlation estimates, for administrative boundary level census-derived socioeconomic status. (PDF 225 kb) [file 12889_2016_3631_MOESM1_ESM.pdf]

Additional file 1

Table S1: Principle Component Analysis results, and inter-item correlation estimates, for administrative boundary level census-derived socioeconomic status

**Socioeconomic variable (n=7)**	**Component loading****	**Corrected inter-item correlation**
Proportion of single-parent families	0.827	0.719
Gross median household income*	0.755	0.639
Proportion of divorced, separated, or widowed among those ≥15 years of age	0.743	0.542
Average value of dwellings*	0.725	0.388
Proportion with no high school diploma, certificate, or degree among those 25-64 years of age	0.699	0.581
Proportion of rented private dwellings	0.654	0.542
Proportion unemployed among those ≥25 years of age	0.508	0.388

Note that all variables were converted to z-scores prior to analysis.
*Reverse coded.
**Kaiser-Meyer-Olkin Measure of Sampling Adequacy=0.78; Bartlett's Test of Sphericity $p<.001$;
Eigenvalue (1st component)=3.51; percent of variance = 50.08%
Internal reliability of socioeconomic status deprivation index (based on sum of all 7 variables): Cronbach's alpha=0.83
